# High Performance Redox Initiating Systems Based on the Interaction of Silane with Metal Complexes: A Unique Platform for the Preparation of Composites

**DOI:** 10.3390/molecules25071602

**Published:** 2020-03-31

**Authors:** Ahmad Arar, Haifaa Mokbel, Frédéric Dumur, Jacques Lalevée

**Affiliations:** 1 Université de Haute-Alsace, CNRS, IS2M UMR 7361, F-68100 Mulhouse, France; ahmad.mh.arar@gmail.com (A.A.); haifaa.mokbel@uha.fr (H.M.); 2 Université de Strasbourg, Strasbourg F-67081, France; 3 Aix Marseille Univ, CNRS, ICR UMR 7273, F-13397 Marseille, France; frederic.dumur@univ-amu.fr

**Keywords:** redox systems, 2K systems, composite materials, free radical polymerization

## Abstract

Currently, Redox Initiating Systems (RISs) of Free Radical Polymerization (FRP) are mainly based on the interaction of aromatic amines with peroxides (e.g., dibenzoyl peroxide (BPO)) that can be both toxic and unstable. In the present work, we aim to replace these hazardous substances in new RIS that can be peroxide-free and amine-free. Our redox two components (2K) initiating system is based on diphenylsilane (DPS) as reducing agent combined with different metal complexes (Mn(acac)_2_, Cu(AAEMA)_2_ or Fe(acac)_3_) as oxidizing agents. For the new proposed RIS, an excellent reactivity is found for the polymerization of benchmark methacrylate monomers under mild conditions (redox polymerization done under air and at room temperature); remarkably, it is also possible to finely control the gel time. Different techniques (optical pyrometry, Real-Time FTIR spectroscopy, Cyclic Voltammetry and Electron Spin Resonance (ESR)) were used to follow the polymerization processes but also to shed some light on the new redox chemical mechanisms.

## 1. Introduction

Redox initiated Free radical polymerization (FRP) is a very well-established technique for polymer production due to its applicability under mild conditions [1,2,3,4,5]. Simply put, the mixing of reducing and oxidizing agents initially separated in two cartridges leads to the formation of free radicals through a redox mechanism to initiate the polymerization process. Such redox systems are already used in widespread research fields: adhesives, composites, biomedical materials, etc. However, the current challenge is to replace the toxic and poorly stable redox agents by safer alternatives [6,7,8,9,10,11]. More particularly, the broadly used dibenzoyl peroxide (BPO)/aromatic amine (e.g., 4-*N,N*-trimethylaniline (4-N,N-TMA)) initiating system is characterized by i) the toxicity issue raised by the use of an amine combined with ii) the issue resulting from peroxides handling and storage and pressing regulations on peroxides. To overcome the aforementioned risks, we propose here new redox initiating systems (RISs) which can be more stable and eco-friendlier [12,13,14,15,16,17,18,19,20,21,22,23,24,25]. Remarkably, these amine-free and peroxide-free systems can be efficient under mild conditions—under air—with controllable gel time. Precisely, diphenylsilane (DPS), selected as a potential reduction agent [26], is originally explored in combination with three oxidative metal complexes (Mn(acac)_2_ or Fe(acac)_3_ or Cu(AAEMA)_2_; with acac = acetylacetonate and AAEMA = methacryloyloxyethylacetoacetate). All these redox agents have great advantages compared to peroxide (e.g., BPO) because they are stable in monomers at room temperature. As an example of the high interest of the new proposed RISs, the polymerization of prepregs (glass or carbon fibers impregnated by organic monomers) is shown for the access to composites.

## 2. Results

### 2.1. Redox Initiating Systems (RISs) using DPS/Metal Complex Combination

**Scheme 1:**
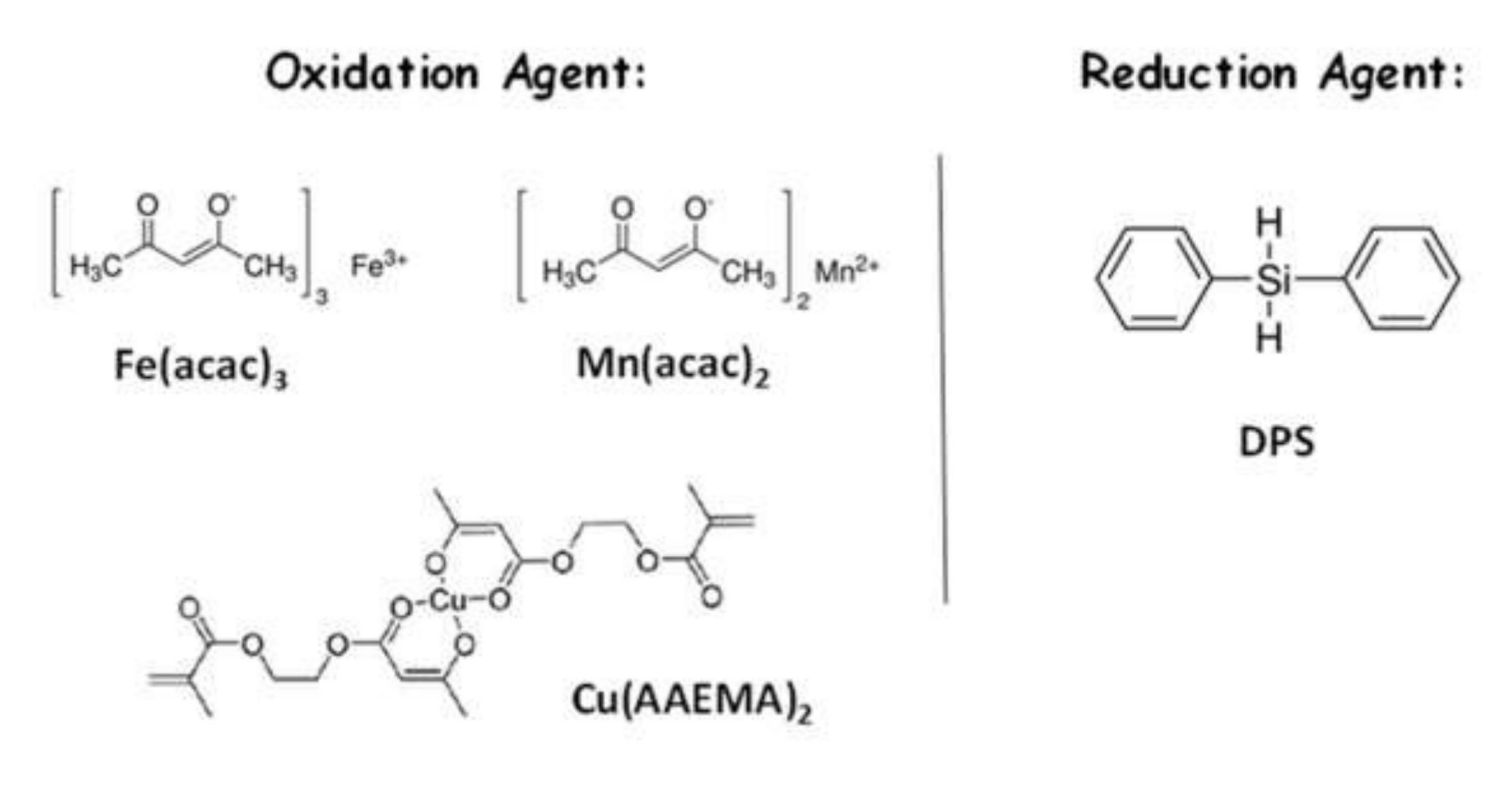
Chemicals used for the redox Free Radical Polymerization (FRP) initiation in this study.

**Scheme 2:**
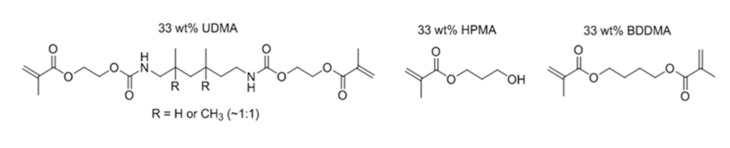
Benchmark methacrylate monomer blend (noted resin 1).

The different chemicals used for the redox initiating systems are gathered in Scheme 1 and the chemical structure of the benchmark methacrylate monomers selected for this study are depicted in Scheme 2. We are interested in polymerization under air; indeed, redox initiating systems are used in many applications under air. Therefore, the resin 1 (Scheme 2), specifically developed in previous works [24,25] (with an adapted viscosity) for polymerization under air, has been used.

**Figure 1:**
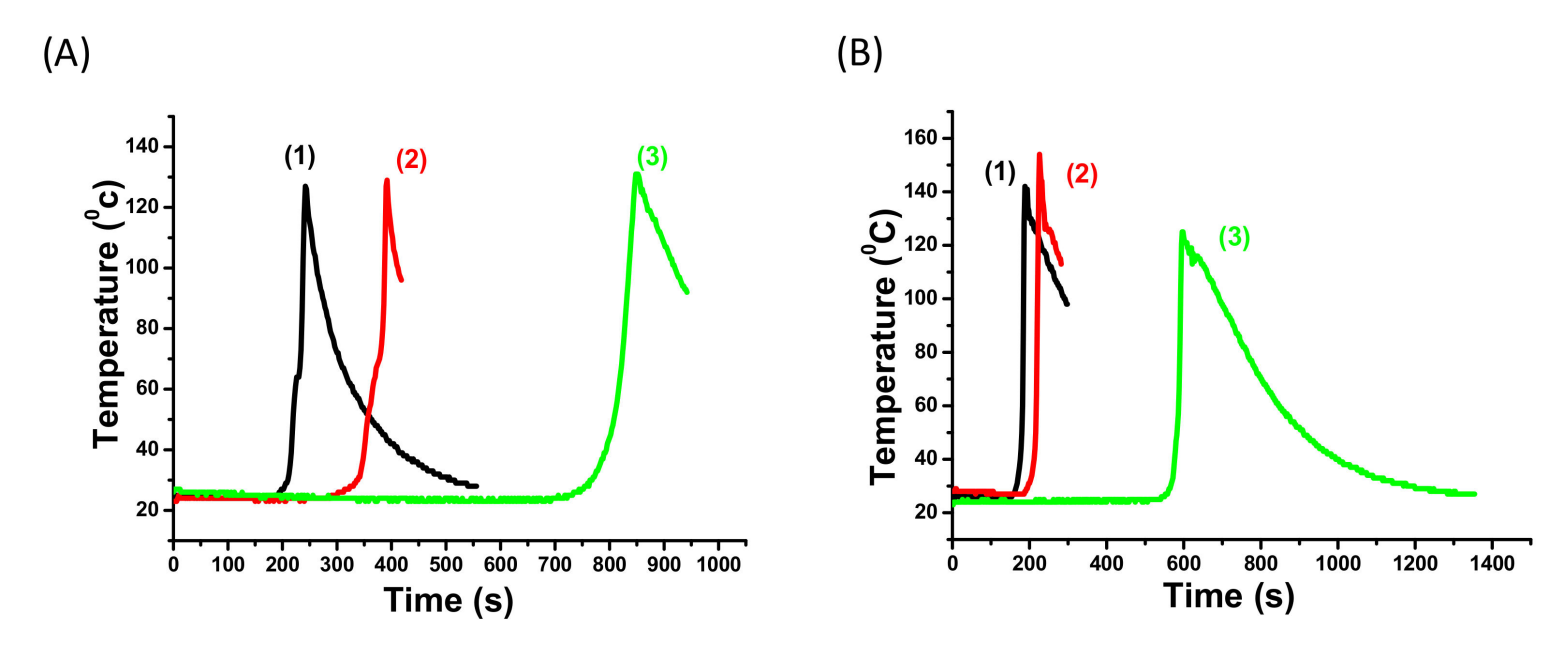
Optical pyrometric measurements (temperature vs. time after mixing; 4 mm thick sample) under air in resin 1 for Redox FRP for (**A**): (1) Cu(AAEMA)_2_/DPS (0.5/2 %wt), (2) Cu(AAEMA)_2_/DPS (1/1 %wt), (3) Cu(AAEMA)_2_/DPS (2/0.5 %wt) and (**B**): (1) Mn(acac)_2_/DPS (0.5/2 %wt), (2) Mn(acac)_2_/DPS (1/1 %wt), (3) Mn(acac)_2_/DPS (2/0.5 %wt) initiating systems.

First, different metal complexes (based on Fe, Cu and Mn) were used in RIS in combination with DPS for the polymerization of the benchmark methacrylate monomer. The polymerization efficiencies of these various redox systems followed by optical pyrometry are depicted in Figure 1. Interestingly, a rather similar reactivity is observed for both metal complexes Cu(AAEMA)_2_ and Mn(acac)_2_ (Figure 1A for Cu(AAEMA)_2_ and Figure 1B for Mn(acac)_2_). A longer gel time is obtained (600–800 s) for a high metal content (~2wt %) which is interesting when a delayed curing (work time) is required. However, for a high DPS content (2% *w/w*), the redox polymerization can be very fast: the gel time decreased by up to 150–200 s. Remarkably, the gel time can be controlled with the concentrations of DPS and metal complexes. Tack-free surfaces are obtained in all cases, showing the ability of these RISs to overcome the classical oxygen inhibition observed for the well-established dibenzoyl peroxide (BPO)/aromatic amine system [24]. The polymerization of methylmethacrylate (MMA) does not work in our conditions; this behavior can be ascribed to the very strong oxygen inhibition conditions (the polymerization is carried out under air) associated with very low viscosity monomers.

4-N,N-trimethylaniline (4-N,N-TMA), a benchmark amine used as reduction agent, does not show any activity in combination with the present metal complexes (no polymerization observed) showing the unique role of DPS for these redox processes.

**Figure 2:**
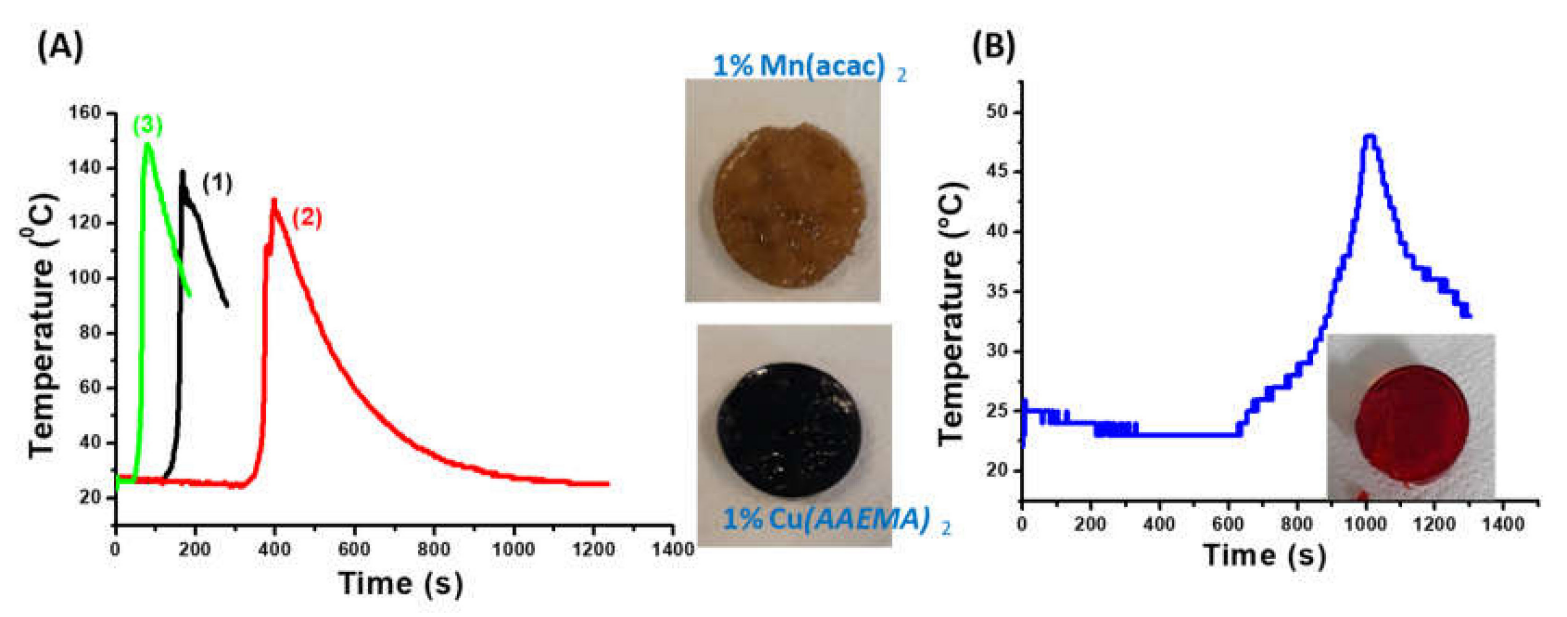
Optical pyrometric measurements (temperature vs. time after mixing; 4 mm thick sample) under air in resin 1 for Redox FRP of (**A**): (1) Mn(acac)_2_/diphenylsilane (DPS) (1/1 %wt), (2) Cu(AAEMA)_2_/DPS (1/1 %wt), (3) Mn(acac)_3_/DPS (1/1 %wt) and (**B**) Fe(acac)_3_/DPS (1/1.4 wt%) initiating system.

The Fe(acac)_3_/DPS system was not found to be very efficient at initiating the polymerization under air. For example, 1000 s were necessary for curing with a lower temperature (only 47 °C, Figure 2B). The results obtained here for Cu(AAEMA)_2_, Mn(acac)_2_ and Fe(acac)_3_ with DPS were compared with those obtained with Mn(acac)_3_ that has been proposed in other redox systems [23] (Figure 2A). Interestingly, Cu(AAEMA)_2_ and Mn(acac)_2_ are characterized by a good efficiency (similar to that observed for Mn(acac)_3_) (Figure 2A). The polymerization parameters for the different redox systems are gathered in Table 1.

**Figure 3:**
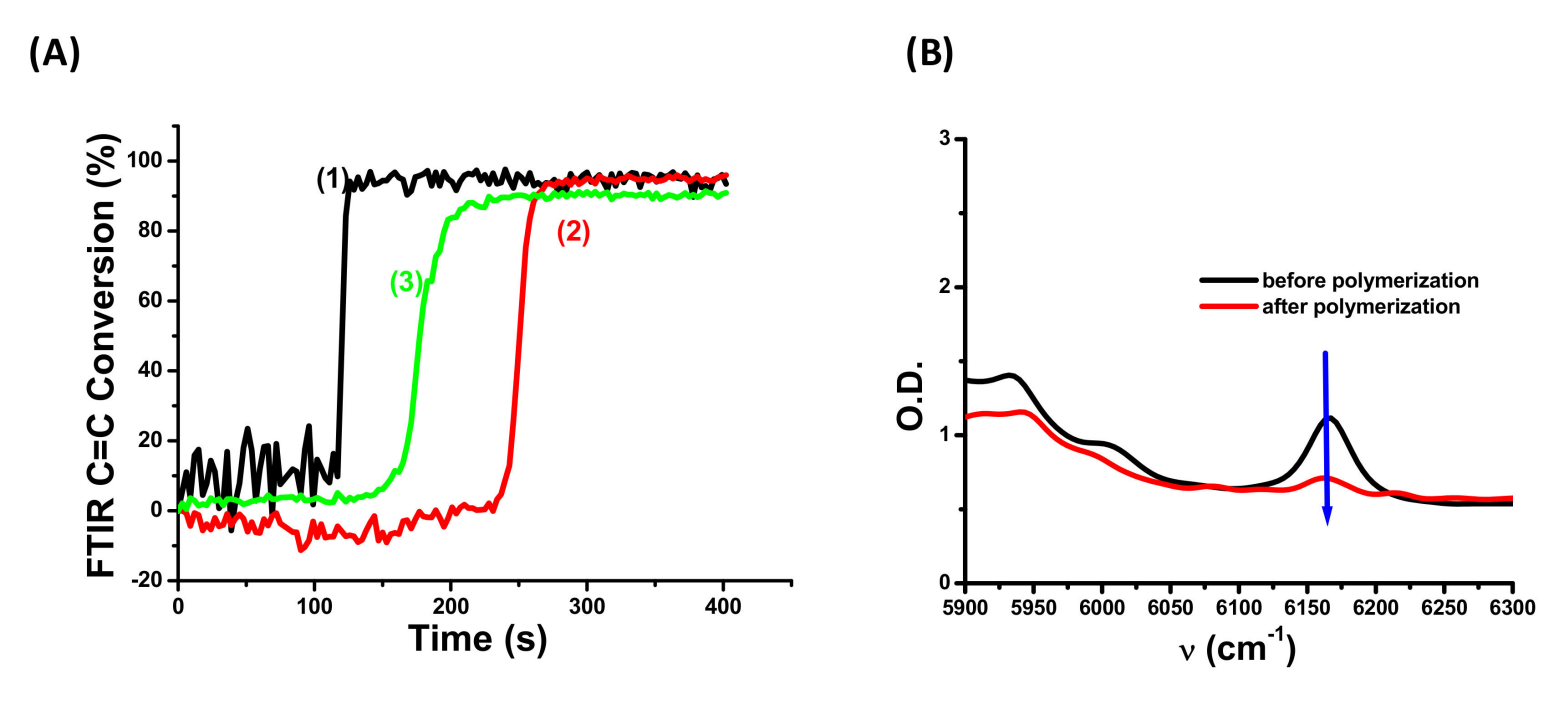
(**A**) Redox Polymerization profiles (methacrylate C=C function conversion vs. time; mixing for t = 0 s) measured in Real-time Fourier Transform Infrared (RT-FTIR) for the resin 1; 1.4 mm thick samples, under air for: (1) Mn(acac)_3_/DPS (1/1 %wt), (2) Mn(acac)_2_/DPS (1/1 %wt), (3) Cu(AAEMA)_2_/DPS (1/1 %wt). (**B**) IR spectra recorded before and after polymerization using Cu(AAEMA)_2_/DPS (1/1 wt %).

These polymerization processes were also followed by Real-Time FTIR spectroscopy. Remarkably, very high final methacrylate function conversions (e.g., FC = 98% - Figure 3A and Table 1) were found under air using the two-component Metal complex/DPS redox systems (1/1 wt %). The recorded FTIR spectra before and after polymerization which showed a full decrease of the characteristic C=C peak at 6160 cm^−1^ are depicted in Figure 3B.

Similar trends are observed between pyrometry and RT-FTIR data. The slight differences of gel times between both techniques can be ascribed to the different thicknesses of the samples (4 mm in pyrometry vs. 1.4 mm in RT-FTIR experiments) leading to different oxygen inhibition behaviors.

### 2.2. Potential Photoactivation of the New Proposed RIS (DPS/Mn(acac)_2_)

**Figure 4:**
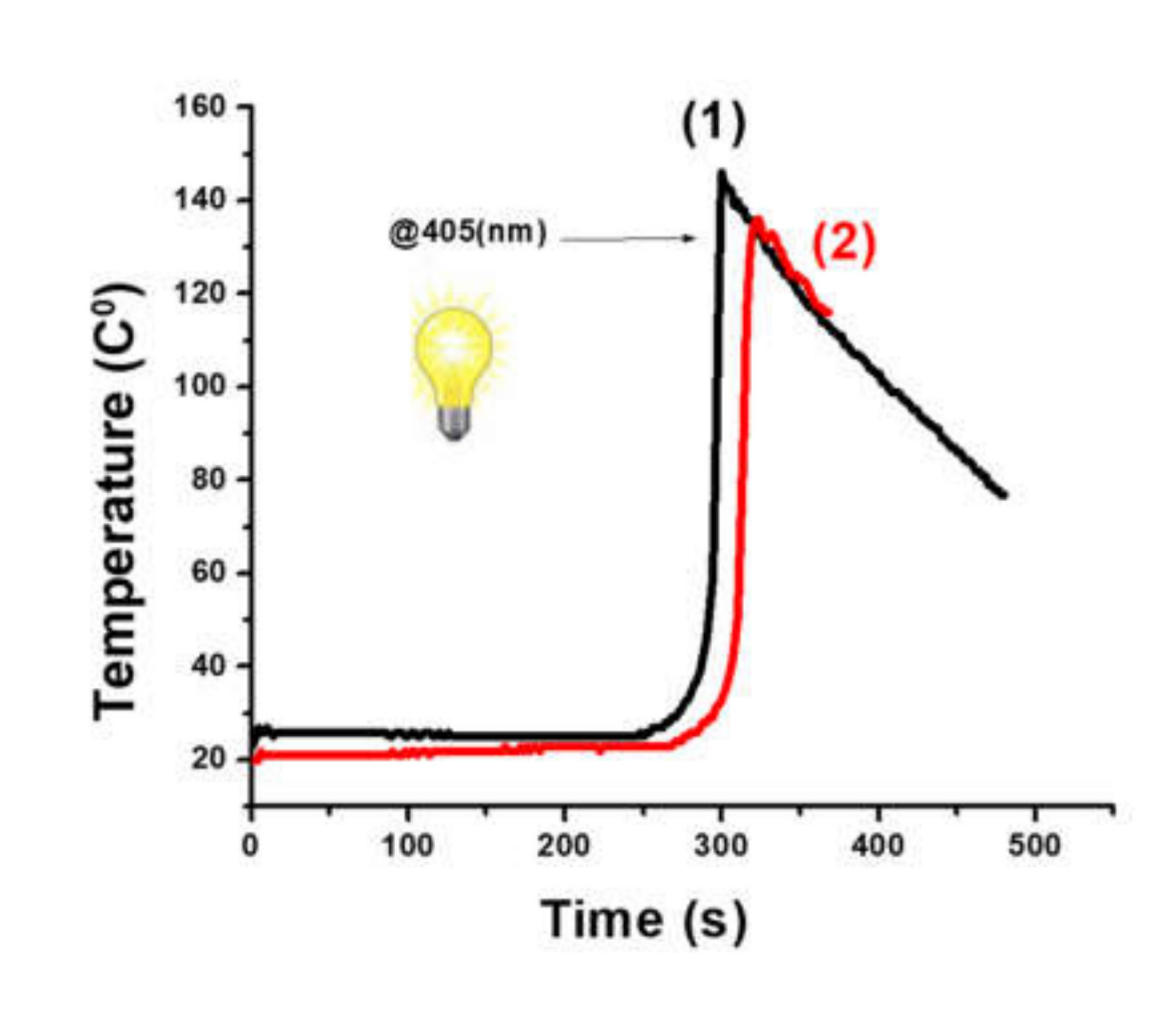
Optical pyrometric measurements (temperature vs. time after mixing; 4 mm thick sample) under air using a Redox Initiating System (RI)S Mn(acac)_2_/DPS (1/1 wt%); (1) with light (LED at 405 nm) and (2) without light.

The possible photoactivation of the RIS was checked upon irradiation with an LED at 405 nm (230 mW/cm^2^). However, it is found that this DPS/Mn(acac)_2_ RIS does not show any significant photoactivation in terms of exothermicity and gel time (Figure 4). This suggests that the photo-induced redox process remains a minor pathway for the FRP compared to the dark redox process. The same behavior is also obtained for Cu(AAEMA)_2_ where no effect of photoactivation is observed upon irradiation.

### 2.3. Stability upon Storage

**Figure 5:**
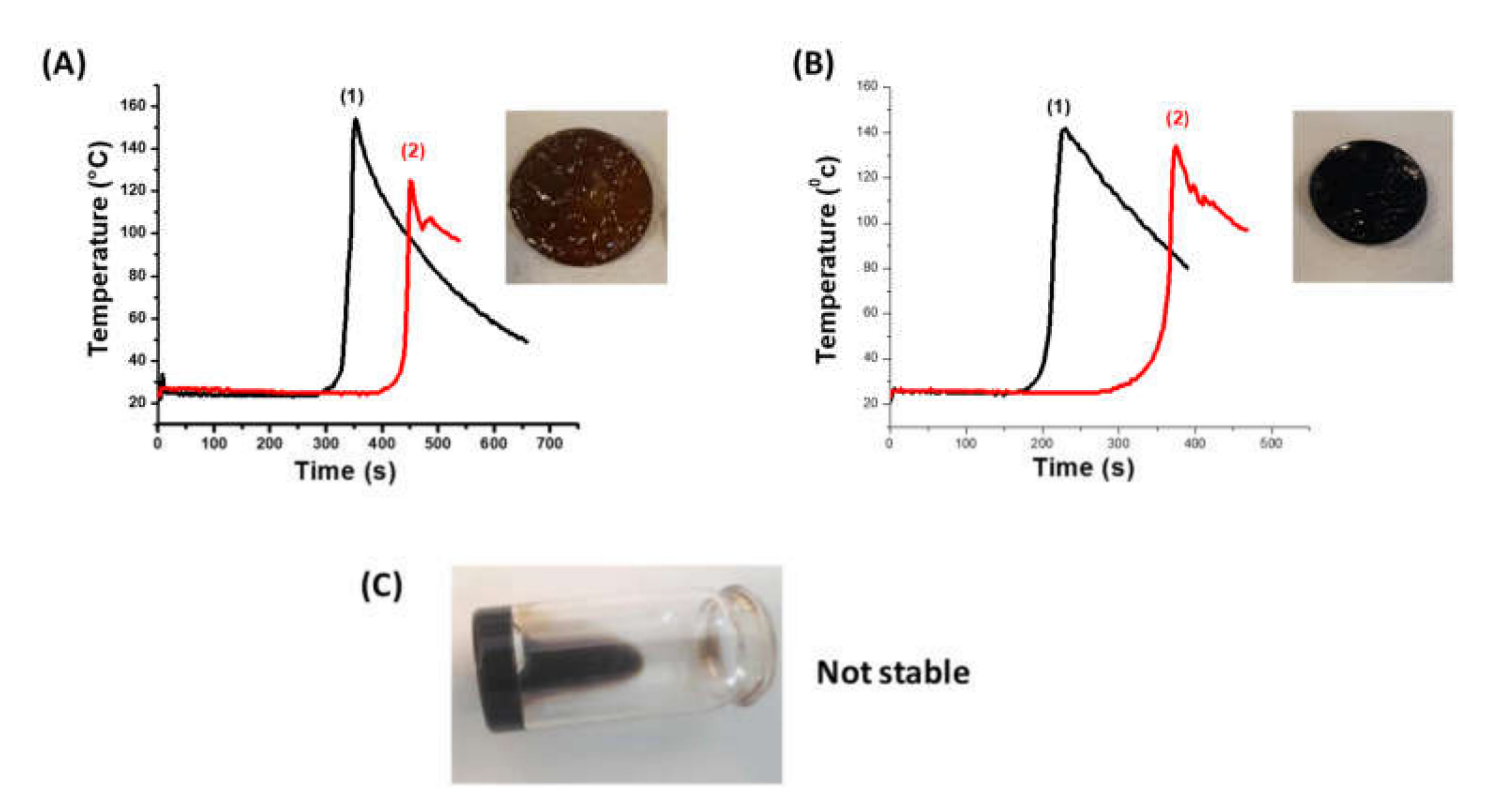
Optical pyrometric measurements (temperature vs. time after mixing, 4 mm thick sample) under air in resin 1 for Redox FRP of (**A**) (1) Mn(acac)_2_/DPS (1/1 wt%) fresh formulations and (2) Mn(acac)_2_/DPS (1/1 wt%) after 6 days of storage at 50 °C; (**B**) (1) Cu(AAEMA)_2_/DPS (1/1 wt%) fresh formulations and (2) Cu(AAEMA)_2_/DPS (1/1 wt%) after 6 days of storage at 50 °C; (**C**) Mn(acac)_3_ (1 wt%) after 48h of storage in resin 1 at 50 °C.

Remarkably, an excellent stability of the proposed RIS was found in monomers. For example, Mn(acac)_2_, Cu(AAEMA)_2_ or DPS formulations showed a stability of 7 days in accelerated aging experiments (when stored in an oven at 50 °C). Interestingly, the gel time is not significantly affected by storage compared to that obtained for fresh formulations (Figure 5). This can extend the areas of application of redox induced polymerization. Mn(acac)_3_ was the best candidate in terms of reactivity (Figure 2) but is not stable in accelerated aging experiments (Figure 5C). In contrast, the other metal complexes (Cu(AAEMA)_2_ and Mn(acac)_2_) showed both excellent reactivity and stability. Therefore, the lack of stability of Mn(acac)_3_ in monomers at 50 °C can limit the use of this metal complex.

### 2.4. Application to the Preparation of Composites

**Figure 6:**
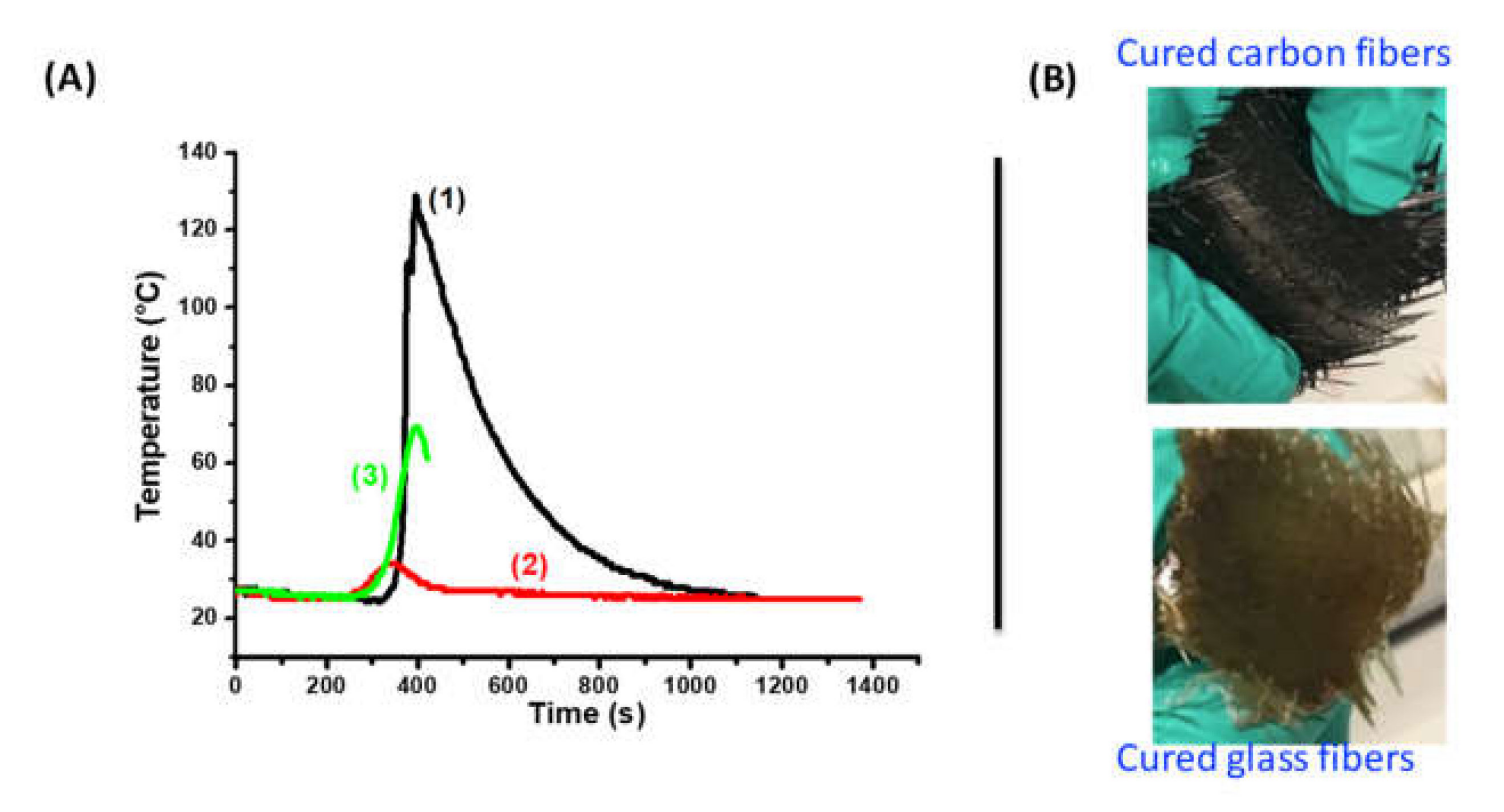
(**A**) Optical pyrometric measurements for the curing of prepregs (glass fibers or carbon fibers/Resin 1 composites) in the presence of (1) Mn(acac)_2_/DPS (1/1 %wt) (without fibers), (2) Mn(acac)_2_/DPS (1/1 %wt) (carbon fibers), (3) Mn(acac)_2_/DPS (1/1 %wt) (glass fibers) and (**B**) Photos for impregnated glass fibers taken after mixing with redox initiating system for 50% glass or carbon fibers and 50% of organic resin.

Finally, we propose to access composites as an application of the new proposed RISs which are characterized by both 1) stability in accelerated aging and 2) highly workable systems (high reactivity and adjusted gel time). Composites materials are highly recommended for different fields as they allow the combination of good mechanical properties with the high flexibility of the polymerization processes. The proposed RISs can fully meet this requirement with a curing time that can be controlled. By curing one layer of prepregs (carbon fibers; thickness ~ 0.2 mm or glass fibers; thickness ~ 0.6 mm) impregnated with organic resin, a good polymerization process is observed when using Mn(acac)_2_/DPS (1/1 wt%). This system is efficient for the preparation of composites where only a few minutes are enough to reach tack-free surfaces (for both the surface and the bottom of the sample) (Figure 6). For composites, lower temperatures are reached as part of the heat released is absorbed by the fibers (Figure 6A).

## 3. Discussion

**Figure 7:**
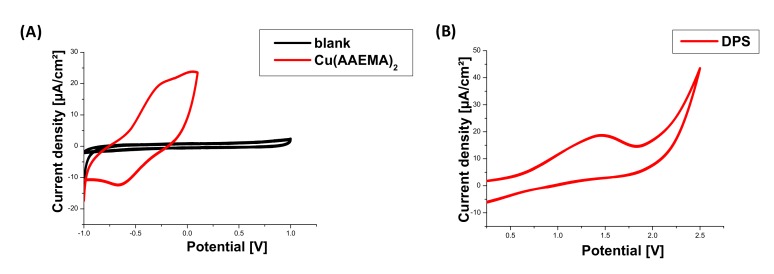
Cyclic voltammetry (potential vs. saturated calomel electrode (SCE)) experiments in acetonitrile (ACN) (containing 0.2 M tetrabutylammonium hexafluorophosphate, 0.1 V/s) for the determination of (**A**) reduction potential of Cu(AAEMA)_2_ and (**B**) oxidation potential of DPS.

In the frame of a redox initiated polymerization, as a general rule, a higher reduction potential of the oxidizing agent (in our case of the metal complex) must lead to a better reactivity. The reduction potentials of the metal complexes follow this order: Cu(AAEMA)_2_ > Mn(acac)_3_ > Mn(acac)_2_ (See Table 1, Figure 7). This order is not in agreement with the experimental trend in redox processes (Mn(acac)_3_ > Mn(acac)_2_ > Cu(AAEMA)_2_) showing that a more complex mechanism than a pure electron transfer occurs for the formation of free radicals (see the ESR results below).

**Figure 8:**
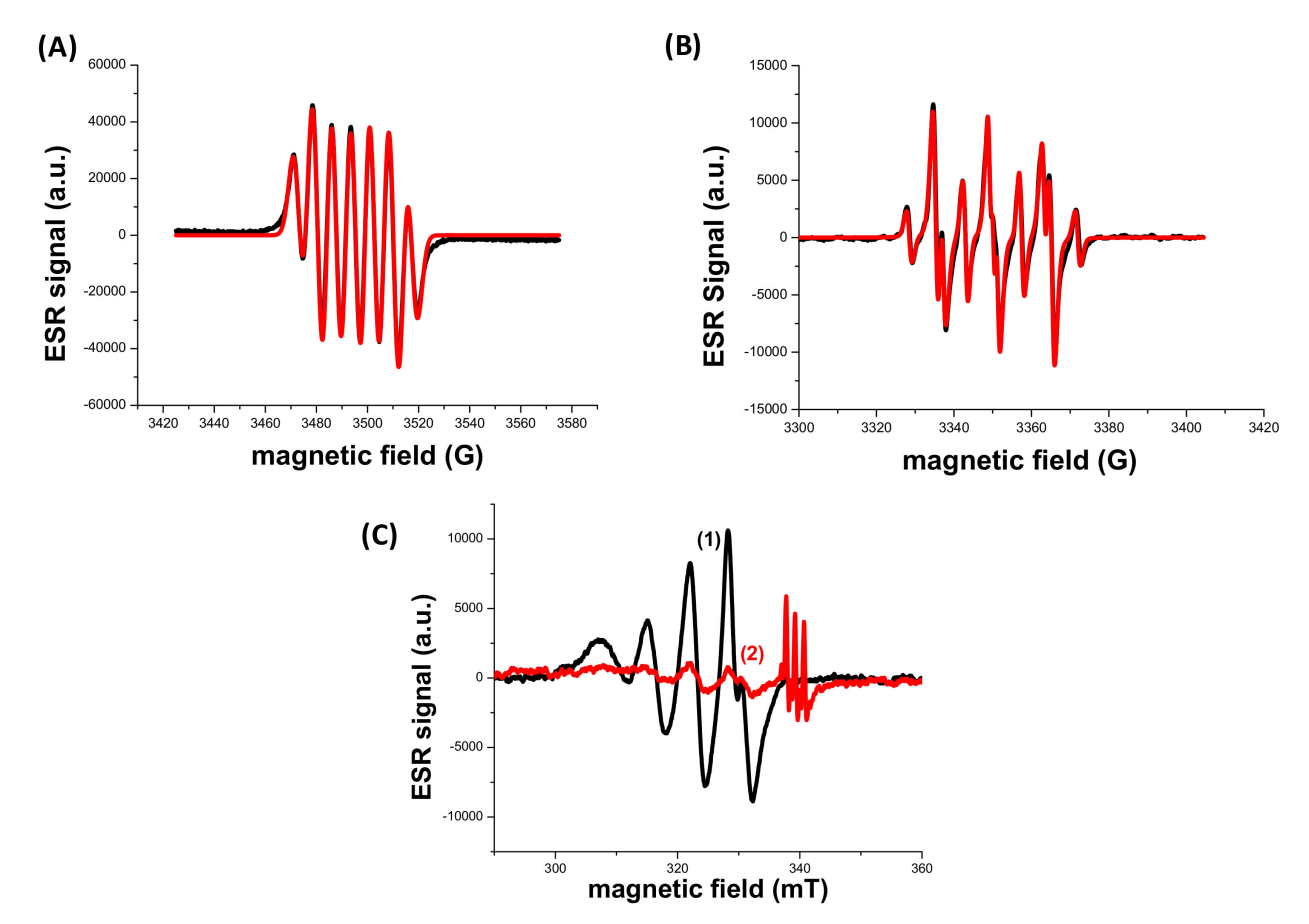
Electron Spin Resonance (ESR)-spin trapping experiment using [PBN] = 10^−2^ M (as spin trap agent); for the mixing of [DPS] = 10^−1^ M with (**A**) Mn(acac)_2_, (**B**) Cu(AAEMA)_2_ simulated (red) and experimental (black) spectra, (**C**) Reduction of Cu (AAEMA)_2_; (1) without DPS and (2) with DPS in tert-butylbenzene; under N_2_.

For a better understanding of the reactivity of redox system, ESR and ESR-spin trapping experiments were carried out for DPS/metal complex systems (Figure 8). Using phenyl-*tert*-butyl nitrone (PBN) as the spin trap agent, free radical adducts are detected after mixing of DPS and Mn(acac)_2_ (Figure 8A) or Cu(AAEMA)_2_ (Figure 8B) in *tert*-butylbenzene and under N_2_. The radical adducts are characterized by the following hyperfine coupling constants: i) aN = 14.8G; aH = 5.3G and ii) aN = 14.9G; aH(2) = 7.4G that can be assigned to the known Ph_2_Si•H/PBN and H•/PBN adducts, respectively [27,28].

This suggests the redox reaction (r1) between DPS and metal complex.

(r1) Ph_2_SiH_2_ + Metal^n^ (acac)_x_ →→→ Ph_2_Si•H + H• + Metal^n−1^(acac)_y_

The radicals generated in (r1) can be assumed as the initiating species for the FRP processes observed above.

More interestingly, for Cu(AAEMA)_2_, the characteristic Cu(II) spectrum (Figure 8C) strongly decreases in presence of DPS (curve 1 vs. curve 2) clearly showing the reduction of Cu(II) to Cu (I) by DPS in agreement with r1. Indeed, Cu(II) can be observed in ESR but Cu(I) is ESR silent.

## 4. Materials and Methods

### 4.1. Chemical Compounds

All chemicals were purchased with high purity and used as received (Scheme 1). Diphenylsilane (DPS) and Mn(acac)_2_ were purchased from TCI-Europe. Fe(acac)_3_ was obtained from Sigma-Aldrich. Cu(II)(methacryloyloxyethylacetoacetate)_2_ will be noted Cu(AAEMA)_2_ and was purchased from Chem Cruz. The efficiency of the different RISs was checked in a benchmark methacrylate monomer blend (noted resin 1) having an adapted viscosity (0.053 Pa.s) and containing 33.3% UDMA (urethane dimethacrylate) 33.3% HPMA (hydroxypropyl methacrylate) and 33.3% BDDMA (butanediol dimethacrylate) (Scheme 2).

### 4.2. Two Cartridges System Used for Redox Experiments

All redox formulations were prepared in bulk in two separate cartridges: a first cartridge with the DPS and the other one containing the Metal complex (Cu(AAEMA)_2_, Mn (acac)_2_ or Fe (acac)_3_). A 1:1 Sulzer mixpac mixer was used to mix the two cartridges at the beginning of each polymerization experiment. As all polymerization experiments were performed at room temperature (~20–25 °C) and under air, an oxygen inhibition can be expected, particularly for the surface in direct contact with air [20].

### 4.3. Redox Polymerization in Bulk Followed by Optical Pyrometry

Optical pyrometry was used to follow polymerization reactions: temperature vs. time profiles were followed using an Omega OS552-V1−6 infrared thermometer (Omega Engineering, Inc., Stamford, CT) having a sensitivity of ±1 °C for 2 g samples (thickness ∼ 4 mm). The redox initiator contents will be given in weight with respect to the monomer in the final blend (wt%). The optical pyrometry is used to check the gel time, i.e., the time required from a two-component mixing to go from a fluid/viscous state to a gel/solid one.

### 4.4. RT-FTIR Spectroscopy

A JASCO 4100 Real-time Fourier Transform Infrared Spectrometer (RT-FTIR) was used to follow the evolution of the methacrylate peak functionality for the thick samples (1.4 mm) in the near-infrared range from 6130 to 6200 cm^−1^.

### 4.5. Electron Spin Resonance (ESR) Spin Trapping (ESR-ST)

Electron spin resonance−spin trapping experiments were carried out using an X-band spectrometer (Magnettech MS400). The radicals were observed at room temperature under nitrogen saturated medium in toluene. *N*-*tert*-butyl-phenylnitrone (PBN) was used as a spin trap agent in a similar way as described in other works [21,22,23,24,25]. ESR spectra simulations were carried out using WINSIM software.

### 4.6. Cyclic Voltammetry

The reduction potentials (E_red_ vs. SCE) of the different metal complexes were determined by cyclic voltammetry in ACN using tetrabutylammonium hexafluorophosphate (0.1 M) as a supporting electrolyte. The procedure was reported in detail in [21,22,23,24,25].

## 5. Conclusions

In this paper, new redox initiating systems are proposed for the free radical polymerization of methacrylate monomers. Contrary to the Mn(acac)_3_, which is not very stable in accelerated aging conditions, both Mn(acac)_2_ and Cu(AAEMA)_2_ showed excellent reactivity and stability. Composites materials based on glass or carbon fibers can be produced using these proposed systems. Other peroxide-free and amine-free systems that can be activated by light will be proposed in forthcoming papers to gather the advantages of photopolymerization and redox polymerization.

## Figures and Tables

**Table 1:** Data of polymerization using different redox systems for the benchmark monomer blend.

System	Gel Time (s)	Maximum T °C	Surface Curing	Final C=C Conversion (%)	E_red_ (V)	ΔG[eV]
Mn(acac)_2_ / DPS 1/1 wt%	110	140	Tack-free	98%	−1.07	2.47
Cu(AAEMA)_2_ / DPS 1/1 wt%	380	130	Tack-free	90%	−0.65	2.05
Fe(acac)_3_ / DPS 1/1 wt%	900	45	Tacky	n.d.	n.d.	n.d.
Mn(acac)_3_ / DPS 1/1 wt%	155	142	Tack-free	98%	−0.85	2.25

n.d.: not determined; ΔG = Eox-Ered (Eox (DPS) = 1.4V), Eox of DPS and Ered of different metal complexes have been determined by cyclic voltammetry (Figure 7).
